# Representation Lost: The Case for a Relational Interpretation of Quantum Mechanics

**DOI:** 10.3390/e20120975

**Published:** 2018-12-15

**Authors:** Raffael Krismer

**Affiliations:** Department of Philosophy, University of Vienna, 1090 Vienna, Austria; raffael.krismer@univie.ac.at

**Keywords:** foundations of quantum theory, entropy maximizing principle, information-theoretic approaches to the quantum state, relational interpretation, toy-models, realism debate

## Abstract

Contemporary non-representationalist interpretations of the quantum state (especially *QBism*, *neo-Copenhagen views*, and the *relational interpretation*) maintain that quantum states codify observer-relative information. This paper provides an extensive defense of such views, while emphasizing the advantages of, specifically, the relational interpretation. The argument proceeds in three steps: (1) I present a classical example (which exemplifies the spirit of the relational interpretation) to illustrate why some of the most persistent charges against non-representationalism have been misguided. (2) The special focus is placed on dynamical evolution. Non-representationalists often motivate their views by interpreting the collapse postulate as the quantum mechanical analogue of Bayesian probability updating. However, it is not clear whether one can also interpret the Schrödinger equation as a form of rational opinion updating. Using results due to Hughes & van Fraassen as well as Lisi, I argue that unitary evolution has a counterpart in classical probability theory: in both cases (quantum and classical) probabilities relative to a *non-participating* observer evolve according to an entropy maximizing principle (and can be interpreted as rational opinion updating). (3) Relying on a thought-experiment by Frauchiger and Renner, I discuss the differences between quantum and classical probability models.

## 1. Introduction

The idea that quantum states do not *represent* (or *correspond to*) physical reality is as old as quantum theory itself. Niels Bohr, e.g., has famously been alleged to assert that “There is no quantum world… It is wrong to think that the task of physics is to find out how nature *is*. Physics concerns what we can say about nature” (attributed to Bohr by Petersen [1] (p. 12)). However perplexing such claims may appear, there still exists a colorful variety of contemporary views that have carried the idea of non-representational quantum states into the 21st century. This paper develops a broad line of defense on behalf of non-representationalist interpretations of the quantum state. In no particular order, the ones I shall focus on are: *QBism* [2,3,4,5,6,7], *neo-Copenhagen approaches* [8,9,10], and the *relational interpretation* [11,12,13]. But even though parts of the argument below might be adopted to suit the purposes of any of these interpretations, I will emphasize the advantages of what appears to me to be the most promising one: a slightly modified version of Rovelli’s relational interpretation. To this end, I present a classical example, which exemplifies how defenders of the relational approach think about quantum theory. Relying on this example, I will be able to discharge several worries that have been levelled against non-representationalists more generally.

Let me start by providing the motivation for this project. The crux of the aforementioned interpretations lies in their commitment to the claim that quantum theory’s probabilistic predictions should be accounted for by information-theoretic means, where the information in question is thought to be relative to some observer. Hence, according to these views, the quantum state is regarded as an irredeemably *relational* concept. While the different interpretations differ substantively over what the quantum state is allegedly relative *to* (i.e., what they mean by the term “observer” -QBism/neo-Copenhagn views: a decision-making agent or subject [2,3,4,5,6], [10] (p. 4); relational interpretation: a physical reference system [11] (pp. 1–2)), this shared commonality runs deep–both from a philosophical but also from a technical viewpoint.

The first set of issues that have plagued non-representationalist views are rooted in the (legitimate) fear that if quantum state ascriptions are observer-relative, objective reality will escape our theoretical clutch. This has both philosophical but also technical dimensions. On the philosophical side, non-representationalists, by virtue of stripping quantum theory of the ability to offer third-person descriptions of the world, have been charged with solipsism or skepticism. Relatedly, the introduction of a subjective element into science has been the source of significant unease. On the technical side, the “psi-ontology” theorems that have emerged in recent years, especially the theorem due to Pusey, Barrett and Rudolph [14] (for an extended review, see [15]), have been interpreted as pulling towards realist interpretations of the quantum state. My first goal will be to ease the pressure that derives from these types of worries. This will be achieved by presenting a classical example of a blatantly non-representational modelling practice which: (1) portrays striking similarities to quantum theory (and the relational way of thinking about quantum theory in particular), and which (2) allows us to demonstrate, using purely classical intuitions, why these arguments against non-representationalism (although *prima facie* plausible) are ultimately guilty of what Dennett once called the “Philosophers’ Syndrome: Mistaking a failure of the imagination for an insight into necessity.” [16] (p. 406).

The second core issue that this paper addresses is more specific, and concerns the question of dynamical evolution. To see why dynamics would play an important role for non-representationalists, notice, first, that an important consequence of their shared commitment to observer-relative states is that the textbook dynamical postulates—von Neumann’s [17] *collapse postulate* and the *unitary evolution* of the Schrödinger equation—are not to be understood in terms of a mechanical/substance-type story of some entity collapsing or evolving. Instead, those changes in the quantum state are to be understood as the process in which the observer rationally updates her opinion—either literally (QBism, neo-Copenhagen), or at least “on the model of” (relational interpretation).

What is thus often cited as a motivation for these views is the analogy between *Lüder’s rule* [18] and its classical counterpart, i.e., *Bayes’ theorem*. [2,19] Lüder’s rule can be taken to justify the idea that one may view von Neumann’s *collapse postulate* as a form of probabilistic conditionalization: the projected (i.e., “collapsed”) state agrees, in its probability assignments to quantum mechanical observables, with the (canonical) generalization of the notion of conditional probability to quantum mechanics. (Recall that in classical probability theory, conditional probability is defined as follows: let P be a probability measure on a Borel field. Then, define a derivative probability measure $P^{\prime }\left(-\right)=P\left(-|A\right)$ for each Borel set A, which is the unique probability measure on the Borel subsets of A such that (1) $P^{\prime }\left(A\right)=1$ and (2) the probability ratios (i.e., the “odds”) are preserved: $\frac{P\left(A\&X\right)}{P\left(A\&Y\right)}=\frac{P^{\prime }\left(A\&X\right)}{P^{\prime }\left(A\&Y\right)}$. From this, Bayes’ theorem can be derived, and this way of thinking about conditional probability can be generalized to the case in which the underlying domain isn’t a Boolean algebra but has the particular structure of an ortho-modular lattice. [20] (pp. 171–175), [21], [22] (pp. 170–173)).

If this analogy between quantum and classical probability models indeed lends credence to non-representationalist approaches, however, we immediately run into a problem: *what is the classical analogue of unitary evolution?* Brown [23], e.g., raises this point in his discussion of QBism. If, for a realist conception of a unitarily evolving wavefunction, the measurement problem was “mysteriously solved” by the projection postulate, then, according to Brown, on the QBist framework it now seems “as if von Neumann’s two motions in quantum mechanics have reappeared in a different guise! The difference now is that the mystery lies with the unitary evolution.” (p. 17) Insofar as unitary evolution appears mysterious on the observer-relative interpretations, this certainly presents challenge: if these interpretations are (at least in part) inspired by the analogy between classical and quantum probability models, is there a classical counterpart of unitary evolution? And if so, can we make sense of it as a form of rational opinion updating?

A central goal of the discussion below will be to show that unitary evolution indeed has a counterpart in classical probability theory. Hence, in *both* cases, classical and quantum, *probabilistic information evolves differently relative to different observers* (such that, as will be shown, the probabilities relative to an external—i.e., “non-participating”—observer are the solutions to an *entropy maximizing problem*). My own relational biases notwithstanding, this is good news for non-representationalists more generally. The presentation of this theorem will follow Hughes and van Fraassen in Ref. [24] (cf. [20]), but since that theorem doesn’t seem to be all that well-known, it is a worthwhile task to repeat it. And while the mathematical details will mirror those of Hughes and van Fraassen, I will: (1) put their theorem in the context of the previously developed example, which will engender certain specific advantages; (2) make explicit in what sense the classical theorem can indeed be viewed as the counterpart of unitary evolution (here I will rely on a result due to Lisi [25]).

The structure of this paper is as follows. Section 2 introduces the terms and intuitions by means of a classical example of a modelling practice that closely resembles the way information is encoded in quantum theory. Section 3 generalizes the initial example to derive, following Hughes and van Fraassen [24], a version of the Schrödinger equation for classical probability models. While Section 2 and Section 3 are intelligible from a purely mathematical perspective, my interpretational motives will be laid bare in Section 4. I will make explicit how the set-up presented in Section 2 and Section 3 instantiates how defenders of the relational interpretation think about quantum theory, and I will also point out several advantages of such an approach. Section 5 makes the transition to quantum theory, where the first goal will be to illustrate how quantum mechanics mirrors the classical way of reasoning presented in Section 2, Section 3 and Section 4. Using a result due to Lisi [25], the analogy between the classical and the quantum case (and the relational approach in particular) will become strikingly clear: by an analogous argument as in the classical case, unitary evolution of probabilities relative to an external observer can be shown to be the result of an entropy maximizing principle (subject to analogous constraints as in the classical case). Having discussed the similarities between classical and quantum probability models, Section 6 will discuss their differences. I argue that a key difference between quantum and classical probability models is that the latter *can*, but the former *cannot* (in general), be supplemented with an ontological story about what the world is like. This will be achieved by placing the previous discussion in the context of a recent thought-experiment proposed by Frauchiger and Renner [26], which can be interpreted to show that, in general, quantum theory has no room for the notion of observer-independent facts.

## 2. The Basic Set-Up

**Figure 1:**
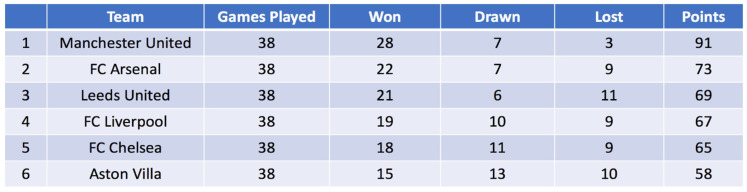
The English Premier League 1999.

To set up the classical example, consider the model of a (presumably) familiar reality that is given in Figure 1:

The specifics of Figure 1 will not turn out to be important, and below I will only discuss the general strategy for how such tables as Figure 1 are produced. Proceeding by means of an example, however, has the advantage of allowing me to introduce the central terminology in intuitive terms.

### 2.1. Measurements

The aim of this modelling practice is to characterize how successful a football team is—we want to create an ordering of the teams on the scale of the natural numbers. This goal will be achieved by letting the teams compete against one another. The games are to be regarded as real processes, real *interactions*, between existing entities (the teams), such that each game has a determinate outcome, which is either *win*, *draw*, or *loss*. Notice that not all combinations of outcomes are possible: although both teams can draw in a single game, it is not the case that both teams can win (or lose). Thus: (1) games are *events*, (2) the outcomes of these events are *definitive* of “what the world is like” (i.e., how the teams will be ranked), and (3) there will be correlations in the descriptions of teams (if one team wins, its opponent must have lost). The set of possible outcomes {win, draw, lose} will be referred to as the “measurement context”; the individual games are called “measurements”. Due to the central importance of these measurement interactions, I will refer to the probabilistic model that results from the considerations below as an “interactional probability model”.

### 2.2. States

Once we have determined a measurement context, we can collect information about the teams. This information will be called a “state.” However, the need will arise to distinguish between different kinds of states, and to introduce different kinds of mathematical structures.

#### 2.2.1. Betting-States

One way to encode information about the teams would be to provide a list of the outcomes of all the individual games. But any attempt to define a relation “…is better than…” by virtue of, e.g., “K is better than L if and only if K has won against L” (information that would be provided by our list) might lead to inconsistencies (if K wins against L, L wins against M, and M wins against K). To achieve our initial aim, of creating a ranking of the teams, we will do much better if we begin by characterizing each team by its total number of wins, draws, and losses. Such a triple of numbers will be called a *betting-state*:

- *Betting-states*. The *betting-state* ascribed to a team is a triple 〈w, d, l〉 where w, d, and l denote the number of wins, draws, and losses respectively.

Clearly, betting-states represent only the outcomes of the games, but not the underlying *mechanisms* by which these outcomes are produced. There may or may not be any systematic way of modelling these mechanism—the point is that we may choose not to worry about such *vastly* complicated things. Hence, we wisely trade descriptive accuracy for predictive success.

Given this definition, it is natural to inquire into the structure of the set of betting-states. Suppose a team is characterized by $\langle w_{1},{\;d}_{1},{\;l}_{1}\rangle$ during the first m games, and by $\langle w_{2},{\;d}_{2},{\;l}_{2}\rangle$ during the subsequent n games. Then, the overall betting-state is given by $\langle w_{1}+w_{2},{\;d}_{1}+d_{2},{\;l}_{1}+l_{2}\rangle$, and hence we can define a component-wise addition for betting-states associated with a single team: $\langle w_{1},{\;d}_{1},{\;l}_{1}\rangle +\langle w_{2},{\;d}_{2},{\;l}_{2}\rangle :=\langle w_{1}+w_{2},{\;d}_{1}+d_{2},{\;l}_{1}+l_{2}\rangle$ (clearly, it makes no sense to add betting-states that are associated with *different* teams). Similarly, we can define a (component-wise) multiplication by a scalar λ: 〈λw, d, l〉=〈λw, λd, λl〉 (where λ might represent the number of rounds in which the same result was obtained, such that component-wise multiplication yields the overall betting-state after λ rounds). Equipped with these operations, the set of betting-states is now a “vector space” (informally speaking, of course; most notably, we are lacking an additive inverse and a multiplicative inverse for multiplication by a scalar).

#### 2.2.2. Odds Comparison

Let’s try and put our betting-states to use. Suppose there is a game coming up—can we use the betting-states to inform our betting behavior? One might, first, propose that betting-states can be used to make *probability assignments* for future events: if a team’s betting-state is given by 〈w, d, l〉 (such that w+d+l=n, where n denotes the total number of games), then the respective probabilities are given as: $p_{\alpha }=\alpha /n$ (α=w,d,l). The suggestion is that these probabilities should guide our betting behavior.

However, there is something a little naive about using the probabilities generated from the betting-states like $p_{\alpha }=\alpha /n$ (α= w,d,l). Here is why: think about a team near the bottom of the league. This team might have a low probability of winning ($p_{w}=\frac{w}{n}<\frac{1}{2}$). But while this may be so, we should be wary not to take this “absolute” description (of a seemingly *intrinsic* property) of the team all that seriously. *Winning*, after all, is inherently relational: you can only win *against* some other team. Suppose that the lowly ranked team plays against a team that is located even further down the table. Its chances of winning, in this case, may actually not be all that low. A sophisticated bettor won’t read too much into the probabilities $p_{\alpha }=\alpha /n$, but will acknowledge that, for each team, the probability of winning, losing, and drawing is correctly specified *relative* to its opponent. Betting behavior, in other words, must be informed by mathematical structures that are sensitive to the measurement that is being performed, rather than those that aim for an “absolute” description of each team.

These ideas can be modelled via what Hughes and van Fraassen [24] (cf. [20] (p. 70)) call an “*odds comparison*.” If two teams are assigned betting-states $\langle w_{1},{\;d}_{1},{\;l}_{1}\rangle$ and $\langle w_{2},{\;d}_{2},{\;l}_{2}\rangle$ (after both teams have played the same number of games), then we may define their *odds comparison* like this: $\langle w_{1}/w_{2},{\;d}_{1}/d_{2},{\;l}_{1}/l_{2}\rangle$. Determining these *relative* odds of two teams (recall that odds, by definition, are probability ratios [20] (p. 69)) will certainly be a most valuable piece of information if we wish to be even moderately sophisticated about our predictions for the outcomes of specific games.

#### 2.2.3. Number-States

Even the odds comparisons, however, aren’t sufficient to unambiguously determine, for any arbitrary pair of teams, which team is better. It is still unclear, e.g., which of the two betting-states (associated with different teams) is better: 〈5, 1, 5〉 or 〈3, 7, 1〉?

A successful way of ordering the teams proceeds by defining a function *s*, which takes as its input a betting-state and assigns to that state a *number*. Depending on whether this number is higher, lower, or equal for the betting-states $\langle w_{1},{\;d}_{1},{\;l}_{1}\rangle$ and $\langle w_{2},{\;d}_{2},{\;l}_{2}\rangle$ that are associated with teams K and L respectively, we will, by definition, know whether K is ranked above, below, or equal to L. These functions will be called *number-state functions*. Let me reemphasize that the primary role of these number-state functions is to assign numerical values to betting-states, which in turn *generates a relative ordering of the teams* on the scale of the natural numbers.

The current number-state function is 〈s(w, d, l)〉=3w+d, where a win is assigned 3 points, a draw is assigned 1 point, and a loss is assigned 0 points. Certainly, there are other sensible options: we could, e.g., define number-state functions “projectively,” such that p(〈w, d, l〉)=w. This would result in a model that would deem only the number of wins to be relevant. Clearly, there are many potential choices of number-state functions, and any such choice is going to be conventional.

This immediately invites the question of how arbitrary our convention is going to be. A natural constraint is that number-state functions should pay tribute to the vector-space structure of the betting-states. Hence, we demand that $s\left(\sum \langle w_{i},{\;d}_{i},{\;l}_{i}\rangle \right)=\sum s\left(\langle w_{i},{\;d}_{i},{\;l}_{i}\rangle \right)$. This is “natural” because we have previously decided to collect, in the betting-states, only information about the outcome of each game, but no information about the *order* of the results.

Before proceeding, let me summarize these remarks by formally introducing two closely related concepts:

- *Number-states*. A *number-state ascription* is an ascription of a numerical value to a betting-state.
- *Number-state functions. Number-state functions* are linear functions from betting-states to the natural numbers.

The reason to insist on this distinction between the number-state functions and the number-states themselves is because one could, in principle, assign numbers to betting-states in an arbitrary way. Thus, it is not trivial to require (as I will) that all number-states derive from a choice of number-state function, but a condition that must be put in by hand.

From the linearity constraint (and the additional constraint that s(〈0, 0, 0〉)=0) we conclude that number-state functions must be of the form $s\left(\langle w,\;d,\;l\rangle \right)=s_{1}w+{\;s}_{2}d+{\;s}_{3}l$. Hence, they can *also* be written as triples $\bar{s}=\langle s_{1},{\;s}_{2},{\;s}_{3}\rangle$, and thus we can (informally, again) consider them to be vectors as well. Number-state functions are therefore of the same mathematical type as the betting-states. The identity of mathematical representations of both types of states, however, should not distract from the fact that they should be interpreted differently.

These observations have two noteworthy consequences. First, notice that if the number-state functions are of the form $s\left(\langle w,\;d,\;l\rangle \right)=s_{1}w+{\;s}_{2}d+{\;s}_{3}l$, we can interpret the resulting number-states (somewhat informally) as being *expectation values* for the overall number of points a team will receive after n games. (Observe that, strictly speaking, this yields an expectation value only if we divide this expression by the total number of games n (the sum of the components): $s\left(\langle w,\;d,\;l\rangle \right)/n=s_{1}p_{w}{+\;s}_{2}p_{d}{+\;s}_{3}p_{l}$, where $p_{\alpha }=\alpha /n$ is the relative frequency of each of the outcomes as specified in Section 2.2.2. Even though these probabilities were previously argued to not be particularly useful (and actively misleading) they are, of course, still probabilities in a mathematical sense, since the frequencies calculated via $p_{\alpha }=\alpha /n$ satisfy the probability axioms.) Secondly, the similarity of mathematical representations of betting-states and number-state functions can be exploited in the following way. The number-states, i.e., the function values s(〈w, d, l〉), can be written in the form of a *dot product* between vectors: $s\left(\langle w,\;d,\;l\rangle \right)=\bar{s}\cdot \bar{x}=s_{1}w+s_{2}d+s_{3}l$. [24] (p. 72).

Before moving on to the main point, which will concern the dynamical evolution of the betting-states, let me add three important remarks:

(1) The correspondence between betting-states and number-states is many-to-one. If, e.g., a team is assigned 15 points after 10 games (by the standard number-state function $\bar{s}=\langle 3,\;1,\;0\rangle$), this is compatible with the team being in betting-states 〈5, 0, 5〉 or 〈4, 3, 3〉 or 〈3, 6, 1〉. In general, therefore, knowledge of a team’s number-state only restricts, but does not determine, which betting-state the team can be said to be in. Although it is natural to say that number-states “encode information about the betting-states,” that information is not fully recoverable from the number-states. (There is common ground here between the football example and the toy-model developed by Spekkens in Ref. [27]: since the number-states put a limit on what can be known about the betting-states, they echo what Spekkens’ refers to as the “knowledge balance principle”, which he introduces as a postulate; cf. [27] (p. 3).) Introducing a further piece of terminology, I will say that a number-state “declares possible” all the betting-states that are compatible with it (so that, e.g., the number-state ascription “K has 15 points after 10 games” declares possible the betting-states 〈5, 0, 5〉, 〈4, 3, 3〉 and 〈3, 6, 1〉 *relative* to the choice of number-state function $\bar{s}=\langle 3,\;1,\;0\rangle$).
(2) The specific number-state assigned to a betting-state has no objective significance, in the sense that (a) the choice of number-state function (from which it derives) is conventional, and (b) the relative ordering of the teams is not preserved under a general change of number-state function. In other words, since different choices of number-state functions (generically) produce different tables, the *relevant* relations— “…is better than…,” “…is worse than…,” and “…is equal to…”—are inherently relative to the choice of number-state function. Thus, these relations cannot be said to *reflect* (or *represent*) objective states of affairs.
(3) Using a piece of terminology familiar from foundational studies on the reality of the quantum state, we would say that the number-states are “ontic” rather than “epistemic” [14,15]. Since this terminology will prove useful again below, it is worth outlining the main idea behind the *ontological models framework*, from which this terminology derives (cf. [15] esp. (pp. 82–88) for a comprehensive overview of the relevant issues). A model is called *ontological*, if each state in the model’s state-space, which will be denoted by Π, corresponds to a classical probability distribution over some measurable space (Λ, Σ) (where Λ is called the *ontic state-space*, and Σ is a Borel (σ-) algebra on Λ). An ontological model is called *ontic* if, for any two states n and m in the state-space Π, every element in the ontic state-space Λ which n declares possible, m declares impossible (this is a somewhat loose, though I hope appropriate, way of paraphrasing the definition given in Ref. [15] for the case in which the elements of the state-space Π don’t ascribe concrete probabilities to the elements of the ontic state-space Λ that they declare possible).

To see why our football example can be regarded as an instance of the ontological models framework, we reason as follows. First, take the betting-states to be elements of the ontic state-space Λ (and let Σ be the standard Borel field obtained from the set of—jointly exhaustive and mutually exclusive—*elementary propositions* “K has won/drawn/lost against L”). The number-states are elements of the state-space Π (which will thus be a subset of the natural numbers). Although the number-states fall short of providing a probability distribution over the betting-states, they declare possible a set of betting-states. However, each of these betting-states is declared possible by exactly one number-state (the last two points follow because, as was noted above, the correspondence between betting-states and number-states is many-to-one). Therefore, number-states are ontic. Despite being ontic, however, there is clearly nothing in the world that “corresponds” to a choice of number-state (since, as was already noted, different choices of number-state functions induce different orderings of the teams). This establishes that number-states are counterexamples to the argument that infers claims about “objective existence in reality” from a state’s *logical* property of “being ontic.” *Number-states are both ontic and non-representational*, and this impairs on the logical validity of the argument that aims to ground representationalist interpretations of the *quantum* state in the recent theorem by Pusey, Barrett & Rudolph [14] (which shows that pure quantum states are ontic).

### 2.3. Dynamics

What is still lacking from our analysis is an account of *dynamical evolution*: we would like to know how betting-states change over time. Consider, thus, how the situation looks *from the point of view of different observers*, who both know that the initial betting-states of some team is 〈w, d, l〉. Suppose that observer A was lucky enough to have acquired a ticket for the ensuing games. Observer B, however, has been less fortunate, and she doesn’t know the outcomes the subsequent games. Since B, unlike A, isn’t collecting any new descriptive information, I will refer to B as an *external* or *non-participating* observer. The question now arises: *how does each observer describe the change in betting-state for a given team?* (Notice that this set-up is the classical counterpart of the Wigner’s friend thought-experiment [28] that will also be discussed below).

#### 2.3.1. How the Situation Looks from A’s Perspective

From A’s perspective, the situation is clear. The correct state to assign after the next game is either 〈w+1, d, l〉 or 〈w, d+1, l〉 or 〈w, d, l+1〉.

#### 2.3.2. How the Situation Looks from B’s Perspective

For B, the situation is more complicated. If B only knows *that* a certain number of games has taken place (i.e., if she agrees with A on the total number of games that were played), but not what the *outcomes* of these games has been, she will have to hedge her bets more carefully. Lacking the relevant descriptive information, she will be left to guesswork and speculation. However, not all speculation is equally good, and in the next section I will outline in what sense B can make a *best* guess (subject to certain constraints) as to what the final betting-state (into which the initial betting-states will have evolved) will be. From here onwards, since B’s best guess is no longer defined as a partial description of the actual outcomes of the games, I will refer to this best guess as the “betting-state relative to B” (or: “B’s betting-state”). This is intended to indicate that the concept of a betting-state functions differently relative to B than it does relative to A.

In formal epistemology, the question of what B’s best guess consists in has been discussed under the heading: “How do probabilities evolve if we do not assume an underlying determinism?” [20] (p. 68) In the next section, this problem will, following the presentation in Ref. [24], be addressed in its most general form. This will lead to a classical version of the Schrödinger equation as the correct equation governing the evolution of B’s betting-state.

## 3. A Classical Version of the Schrödinger Equation for Optimal Opinion Updating Relative to Non-Participating Observers

Before we can address B’s dynamics problem, it will be useful to first generalize the situation described thus far. Box 1 summarizes the kinematics of interactional probability models, for which Section 2 gave a specific example.

Box 1 **Kinematics of Interactional Probability Models.**

**Kinematics of Interactional Probability Models**

1. **Measurement Context:** In the general case, our *measurement context* consists of n distinguishable outcomes of the interactions between the entities within the model’s scope.
2. **Betting-states relative to B**: The *betting-state relative to B* is an n-tuple of numbers $\bar{x}=x_{1},\;\ldots ,{\;x}_{n}$, the components of which specify B’s best guess for how many times a particular outcome was observed (this best guess will be a *true* guess, if B happens to know the outcomes of the games). The set of betting-states has the following structure:
  - *Betting-states are vectors*. Betting-states can be added (component-wise) and multiplied by a scalar (component-wise).
  - *Odds-Comparison:* The *odds comparison* of two betting-states $\bar{x}=\langle x_{1},\;\ldots ,{\;x}_{n}\rangle$ and $\bar{y}=\langle y_{1},\;\ldots ,{\;y}_{n}\rangle$ is defined (for $\sum x_{i}=\sum y_{i}$) as $\bar{x}/\bar{y}:=\langle x_{1}/y_{1},\;\ldots ,{\;x}_{n}/y_{n}\rangle \;$(if well-defined).
3. **Number-states**: A *number-state ascription* is an ascription of a numerical value to a betting-state. All number-states that will be considered are required to arise from a choice of number-state function.
  - *Number-state functions. Number-state functions* are linear functions from betting-states to some choice of number field. Thus, number-state functions are characterized by n numbers, $s=\langle s_{1},\;\ldots ,{\;s}_{n}\rangle$, such that: $s\left(\bar{x}\right)=\sum s_{i}x_{i}$. Hence, they are vectors of the same mathematical type as the betting-states.
  - *Number-states are expectation values*. From the point of view of interpretation, the equation $\langle \bar{x}\rangle =s\left(\bar{x}\right)=\sum s_{i}x_{i}$ yields an *expectation value* (for the number of points associated with a team after a certain number of games, for caveats, cf. Section 2.2.3).
  - *Number-state functions are coordinatizations*. Since the primary role of number-state functions is that of generating an ordering of the teams, I will say that number-states are “coordinatizations” of the betting-states (cf. Section 4).

Let’s return to the problem of dynamical evolution (from the point of view of an external or non-participating observer B). Suppose that B knows the initial betting-state $\bar{x}\left(0\right)$ of a team, at time t=0, and wishes to update her state to a final time $t_{f}$, yielding a state $\bar{x}\left(t_{f}\right)$. The question we are facing is this: what is B’s best guess for the final betting-state $\bar{x}\left(t_{f}\right)$?

Before she can even begin to address this question, B must make an initial assumption. This concerns that fact that so far, the components of the betting-states were integers. However, since B is now confronted with the problem of having to account for the *changes* that occur in the components of her betting-states, and since it can be computationally very difficult to model discrete changes, she will do well to transform her problem into one that can be handled more easily. This can be achieved by allowing the components of her betting-state to evolve *continuously* (with respect to a parameter t). Therefore, she will embed (in the sense of providing an injective structure-preserving map) the betting-states—which are of the form 〈w, d, l〉 (with the components elements of the natural numbers)—into the set of triples of the form 〈w, d, l〉 (where the components now lie in the *real* numbers). This embedding is simply the trivial embedding of the natural numbers into real numbers (i.e., the identity map: id:a⟼a). The reason why this assumption is justified, is because the embedding preserves the relevant algebraic structure (in particular: the vector-space structure and the odds comparison). Therefore, although the problem has now been transformed, it has not been significantly altered. [24] (p. 852) In a slight abuse of terminology, I will still refer to these new states as “betting-states relative to B”.

In this more general scenario, we can ask what types of constraints should be respected, for the evolution of $\bar{x}\left(0\right)$ to $\bar{x}\left(t_{f}\right)$. One reasonable constraint is that dynamical evolution shouldn’t mess up the odds comparison of the teams. Otherwise, the evolution would (unnaturally) privilege certain teams over others, which might distort—not the facts, mind you!—but what is rational to believe from the point of view of observer B. To implement this, we will need the concept of a symmetry.

- *Symmetry*. A *symmetry* of the space of betting-states is a linear map U that maps the space onto itself such that the odds comparison of betting-states is preserved: $U\left(\bar{x}\right)/U\left(\bar{y}\right)=\bar{x}/\bar{y}$ (if well-defined). [24] (p. 857), [20] (p. 72).

Our first constraint, that odds comparisons should be preserved, therefore becomes: B’s evolved state should be the result of a symmetry transformation on her initial state.

Before proceeding, let me add some brief comments. (1) As an important observation, notice that Bayes’ theorem is also derived from a symmetry condition (this is a key element that Hughes and van Fraassen in Ref. [24] draw attention to). (2) Notice that the justification for the first condition relies on the *relational nature* of our description—what we want to be preserved is a relational quantity (the odds comparison) rather than the quantities $p_{\alpha }=\alpha /n$ (α= w,d,l), which characterize a *single* team. (3) We will have a chance to use the following theorem:

**Theorem.**  *A transformation U is a symmetry if and only if there exist positive real numbers* $u_{1},\;\ldots ,\;u_{n}$ *such that* $U\left(\bar{x}\right)=\langle u_{1}\;x_{1},\;\ldots ,u_{n}\;x_{n}\rangle$ *. [24] (p. 857), [20] (pp. 71–73).*

**Proof.**  See Appendix A. □

The next condition is that evolving by a time $t_{1}$ and then by $t_{2}$ should be the same as evolving by $t_{1}+t_{2}$. (“The set of evolution operators form a semi-group”). This gives rise to another definition:

- *Uniform motion*. A *uniform motion* on the space of betting-states is an element of the set $\left\{U\left(t\right),\;t\geq 0:U\left(t_{1}\right)\circ U\left(t_{2}\right)=U\left(t_{1}+t_{2}\right)\right\}$ of symmetries labelled by a continuous parameter t. [24] (p. 857), [20] (p. 72).

There is another straightforward theorem that will prove useful:

**Theorem.**  *If two betting-states* $\bar{x}\left(0\right)$ *and* $\bar{x}\left(t\right)$ *are related via a uniform motion* $\bar{x}\left(t\right)=U\left(t\right)\left(\bar{x}\left(0\right)\;\right)=u_{1}\left(t\right)\;x_{1}\left(0\right),\;\ldots ,u_{n}\left(t\right)\;x_{n}\left(0\right)$ *, then there exist positive real numbers* $k_{1},\;\ldots ,\;k_{n}$ *such that* $u_{i}(t)=\;e^{k_{i}t}$ *. [24] (p. 858), [20] (pp. 72–73).*

**Proof.**  See Appendix A. □

The next constraint will put on the breaks. We still don’t know anything about the coefficients $k_{i}$ that figure in the previous theorem. If those are chosen at random, the final state $\bar{x}\left(t_{f}\right)$ might be arbitrarily distant from the initial state. To prevent this, which we should, if B’s guess is to be taken as a “best” guess, we impose that the *overall* change induced by the evolution operators is minimal. Now, the total rate of change in B’s betting-state is given by: $\frac{\partial }{\partial t}\sum x_{i}\left(t\right)=\sum k_{i}x_{i}\left(t\right)$. Furthermore, since the $k_{i}$’s are positive real, we know that all derivatives $\frac{\partial^{\left(m\right)}}{\partial t^{\left(m\right)}}\sum x_{i}\left(t\right)$ are positive real. Thus, in particular, the second derivative will be greater than 0, which means the first derivative will be a monotonically increasing function. To minimize the overall change in B’s betting-state, we can therefore require that the quantity $\frac{\partial }{\partial t}\sum x_{i}\left(t\right)=\sum k_{i}x_{i}\left(t\right)$ be minimal for $\bar{x}\left(t_{f}\right)$. Notice that, since $k_{i}\sim \ln\left(\frac{x_{i}\left(t_{f}\right)}{x_{i}\left(0\right)}\right)$, this has the form of an *entropy maximizing condition*. [24] (pp. 858–860).

There are two final conditions. First, we want the total number of games to be known: the betting-state relative to B should be *normalized*, in the sense that B knows the total number of games that have been played at the final time: $\sum x_{i}\left(t_{f}\right)=n\left(t_{f}\right)$. Secondly, we impose that the number-state (which has played no role so far) at the final time is fixed, i.e., that $\sum s_{i}x_{i}\left(t_{f}\right)=r\left(t_{f}\right)$ (thus $r\left(t_{f}\right)$ denotes the final number-state of the team). The interpretational spin we could put on this is that the number-states assigned to the teams should be *the same* for both observers. Hence, we allow that A communicates to B (after all games have been played) what the final number-state of the team is. Since there might be many different betting-states that give rise to the same number-state, this condition plays an important role: the allowed betting-states are those that the number-state declares possible (in the terminology from Section 2.2.3).

Summarizing the discussion, we can now present the problem of B’s dynamical evolution as the following *optimization* problem:

Box 2 **Non-participating observer B’s dynamical problem.**

**Optimal Rational Opinion Updating Relative to Non-Participating Observers**

*B’s dynamical problem*: Find a set of evolution operators U that relate the betting-states $\bar{x}\left(0\right)$ and $\bar{x}\left(t_{f}\right)$ (at time 0 and t_f_ respectively) such that:

1. U is a uniform motion: $\bar{x}\left(t_{f}\right)=\;U\left(t_{f}\right)\left(\bar{x}\left(0\right)\right)$.

According to the theorems mentioned above, this means that we already know that $\bar{x}\left(t_{f}\right)=\langle u_{1}\left(t_{f}\right){\;x}_{1}\left(0\right),\;\ldots ,u_{n}\left(t_{f}\right){\;x}_{n}\left(0\right)\rangle$, and that there exist positive real numbers $k_{1},\;\ldots ,{\;k}_{n}$ such that $u_{i}\left(t_{f}\right)={\;e}^{k_{i}t_{f}}$.

2 Find real numbers $k_{1},\;\ldots ,{\;k}_{n}$ such that $\sum k_{i}x_{i}\left(t_{f}\right)\to \min$. This is subject to the constraints that:
  - The final betting-state relative to B is normalized: $\sum x_{i}\left(t_{f}\right)=n\left(t_{f}\right)$.
  - The final number-state is agreed upon by both observers A and B: $\sum s_{i}x_{i}\left(t_{f}\right)=r\left(t_{f}\right)$.

It now can be proven that [24] (pp. 860–862):

**Theorem.**  *There exist constants v & w such that the*$u_{i}\left(t_{f}\right)$*’s are given by*$u_{i}\left(t_{f}\right)=\;e^{vt_{f}}\;e^{ws_{i}t_{f}}$.

**Proof.**  See Appendix A. □

In other words, *B’s optimal opinion change is given by something that looks a lot like a classical version of the Schrödinger equation:* the constant w plays the role of Planck’s constant and the $s_{i}$’s play the role of the eigenvalues of the Hamiltonian. w and v are Lagrange multipliers that are uniquely determined by the boundary-conditions 2a/b in Box 2. [24] (p. 861) Observe, also, that evolution depends on a choice of number-state function; hence different such choices induce different “shifts” in different “bases.” Therefore, the final betting-state relative to B—which will be a different state than the betting-state relative to observer A—is uniquely fixed by the above conditions. Therefore, the evolution of B’s betting-state is *deterministic*.

This concludes the mathematical discussion. The challenges ahead, of course, are still quite significant. For one, we haven’t said anything about quantum mechanics yet. While the above theorem certainly resembles, rather closely, the form of the solutions to the Schrödinger equation, there are still important differences (most notably, the additional factor of $e^{{\mathrm{vt}}_{f}}$ and the absence of the imaginary unit *i*). Section 5 tries to substantiate the claim that the above theorem can indeed be viewed as a classical version of the Schrödinger equation. Specifically, I will argue that it is the form of the *problem*, as well as the form of the *solution*, that justifies viewing the result of the above theorem as the classical analogue of the Schrödinger equation. To pave the way for this discussion, Section 4 discusses some conceptual subtleties that, so far, haven’t received the attention they deserve. (There is another worry, unrelated to the subsequent discussion, to which an anonymous referee has alerted me. This concerns the fact that that the theorem predicts that the total number of games n(t)—i.e., the sum of the components—evolves as a sum of exponential functions, which might appear counterintuitive. Why would B conclude that n(t) evolves in this fashion (as opposed to, say, making the more reasonable assumption that events occur at a constant rate)? To see that the theorem produces “reasonable” results, there is an explicit example worked out in Appendix B.).

## 4. Preliminary Discussion—Some Advantages of the Example

The setting I have chosen will likely have struck the reader as somewhat peculiar. At the very least, this *interactional probability model* is quite detached from the paradigm cases of probabilistic models, such as coin-flipping and dice-tossing, which still often guide our thinking about these matters. Here, I argue that this is a good thing, which will be achieved by locating the example relative to some familiar issues in the philosophy of probability. I will also expose the sense in which Rovelli’s relational interpretation [11,12,13] (or: at least one potential version of it; cf. Section 5) is inspired by such interactional probability models as the one that was presented above. Using the classical example, I will try to illustrate why the relational view might enjoy some important advantages over its closest neighbors (such as QBism or neo-Copenhagen views).

Now, if we had chosen a more paradigmatic setting, such as a coin being tossed, the theorem that fixes the evolution of B’s probabilities would still have gone through. After all, Hughes & van Fraassen’s presentation in Ref. [24] can be interpreted in this way: how do the probabilities of a *single* coin, or a dice, evolve relative to different rationality constraints (which specify the epistemic situation of different observers: observer A has knowledge of *outcomes*, while observer B only knows *that* the coin was tossed)? For this reason, proponents of observer-dependent interpretations of the quantum state other than the relational view might wish to include the result of Section 3 in their argumentative toolbox. Nevertheless, the set-up from Section 2 and Section 3 has some strong conceptual advantages over the more traditional examples, which merit closer inspection.

### 4.1. The Betting-States are not “Absolute Descriptions”

The claim that the betting-states are not “absolute” descriptions of the teams is intended in the following sense: had we chosen a more traditional setting of a coin being tossed, the possible outcomes *heads* and *tails* certainly characterize fully *intrinsic* properties of the coin. But while the concepts of *winning*, *losing*, and *drawing* may give away the impression of characterizing the teams “absolutely”, this is only an appearance: you can only win, lose, or draw *against* another team. There are two noteworthy aspects to this.

#### 4.1.1. Betting-States are Correlations

The first remark relates back to the observation that there will be *correlations* in the betting-states. After each round, as many teams will have one more win as there will be teams with one additional loss. Hence, if you ask a team how they played, and they tell you that they have won, you know—instantaneously and without hesitation—that the other team has lost. For that reason, the above example exemplifies a central commitment of Rovelli’s relational view, namely that the ascription of any particular state (which is interpreted as codifying observer-dependent information) is equivalent to asserting that there exists a certain *correlation* between systems: “*The fact that the pointer variable in O has information about S (has measured q) is expressed by the existence of a correlation between the q variable of S and the pointer variable of O.*” [11] (p. 9).

This is to be contrasted, in particular, with neo-Copenhagen interpretations of especially Zeilinger and Brukner [8,9], according to which quantum theory is also grounded in information-theoretic considerations, but in a different sense. Zeilinger [8], e.g., postulates his “foundational principle”, which states that each elementary system carries one bit of information. This, clearly, suggests that the amount of information somehow characterizes an intrinsic property of certain systems, leading to the vexing claim that “information is physical” (cf. Timpson in Ref. [29] (pp. 67–73; 152–158) for a pointed discussion of the problems associated with such claims). On the relational view, information is simply the existence of correlations, and hence, this view sidesteps these types of debates (of whether we can make coherent sense of the suggestion that information is physical). This advantage of the relational view over the neo-Copenhagen approach (one of its closest allies) carries over to the second point I would like to stress.

#### 4.1.2. “How Things Are” vs. “How Things Affect One Another”

It is important to observe that the betting-state relative to B isn’t useful because it characterizes “how physical systems are.” On the contrary, it is useful primarily because it determines the odds for how a team will fare when playing against some other team (since we required the odds comparison to be preserved). To put it in a slogan: B’s problem is all about “how things affect one another” rather than about “how things are.” In that sense, this example perfectly instantiates what is perhaps *the* core commitment of Rovelli’s relational view: “The core idea is to read the theory [of quantum mechanics] as a theoretical account of the way distinct physical systems *affect each other* when they interact (and not the way physical systems ‘are’).” [12] In other words, on the relational interpretation, quantum theory is interpreted along the following lines: from the previous point, we know that quantum state ascriptions are the result of preparation procedures, in which a correlation between measurement apparatus and system has been established. [11] (pp. 9–10) At the same time, the point of collecting information in this way is that this allows predictions to be made (from the point of view of different observers) for the outcomes of possible future interaction with other systems (as opposed to: preparation procedures playing the role of characterizing “how quantum mechanical systems are”).

### 4.2. Completeness

The football example is “universal,” in the sense that the interacting entities (the teams) are, albeit *physically* distinct (since they are characterized by different betting-/number-states), *metaphysically* equal. This is important for the following reason. It is uncontroversial, though easily overlooked in paradigmatic cases of probabilistic processes (such as a coin being tossed), that probabilities are a peculiar mixture of being both “absolute” descriptions of “how things are,” but also descriptions of “how things affect one another,” at least in the following sense: suppose, e.g., that we want to toss a coin, but that this coin is also a magnet, such that if it is tossed over a sufficiently strong magnetic field, it will always show *heads*. Since we might, e.g., turn the magnetic field on and off, the probabilities that are to be assigned for each toss should always be thought of as functions of: (1) of the coin itself, but *also* (2) the environment with which the coin, once tossed, will interact. In the traditional settings, however, the environment will generically obey *different* (perhaps non-*probabilistic*) laws than the coin itself (which is modelled probabilistically). In that sense, although both environment and system may obey well-defined sets of physical laws, they will have to be viewed as metaphysically distinct. This situation changes in the football example. The model universally applies to all entities within its scope, and this shows how a theory of “how things affect one another” can consistently held to be *complete*. We simply don’t require any story about “how things are” in order to have a substantive and powerful account of “how things affect one another.” And this, in turn, captures the intuition behind why Rovelli’s relational interpretation aspires to be *complete:* on the relational view every system will be treated as a *quantum mechanical* system; all systems will be metaphysically equal, though physically distinct, partners in the interactions, and the description in terms of *transition probabilities* (from earlier to later states) will be held to be *complete*. [11] (p. 7), [11] Since completeness is a clear virtue of any interpretational strategy, the relational view will enjoy this advantage over its next-door neighbors such as QBism or the neo-Copenhagen view, which require further resources to describe reality (QBists in terms of direct experience [7]; neo-Copenhagens in terms of classical mechanics [10]).

### 4.3. The Role of Number-States

The football example reserves an explicit role for the number-states, which deepens the analogy to quantum states in at least two ways.

#### 4.3.1. Number-States are Expectation Values

Notice, first, that we can construct a counterpart to the problem of “choosing a number-state function” for the traditional example of a coin being tossed. For example, since we could associate any arbitrary pair of numbers with either side, we could, given any such assignment, calculate a numerical expectation value (just as in the football example). And this expectation value is also going to be conventional (as the numbers we could choose to associate with each side might be selected at random). First of all, this would be somewhat artificial. Secondly, this is beset with some vexing philosophical problems. For suppose we have decided to label the different sides of the coin with the numbers 1 and 2. In the next step, we will have to ask ourselves whether there is a correct way of labelling the different sides of the coin. If the coin is slightly biased or asymmetrical, which must be assumed for any non-ideal case, then it makes a difference which number we assign to which side. But we can’t know what that bias is, if we don’t already know what the correct labelling consists in (arguing that there is a primitive labelling of *heads* and *tails* only pushes the problem one step back). In the football example, these problems, which echo some traditional problems from the philosophy of probability, simply disappear (which, of course, is due to the fact that in the football example, symmetry considerations don’t concern any intrinsic property of a single team, but a relational quantity, i.e., the preservation of the odds comparison).

#### 4.3.2. Number-States are Coordinatizations

The number-states have the peculiar role of generating a *relative ordering* of the teams (as, e.g., the relation “better than” was induced by a choice of number-state). This has an obvious counterpart in physics: the claim that a particle is *located at position x*, must always be relative to some reference system (which defines our origin). Hence, physics is in the same business of *coordinating systems* (a) relative to other systems and (b) relative to a choice of coordinates (“S is m meters to the left of O” maps onto “K is n points better than L”). Notice, in particular, that the condition that number-states were agreed upon by both A and B (which implies that the ordering of the teams is the same for both observers) played an important role in the derivation of the equation that governs the evolution of B’s betting-states.

## 5. Quantum vs. Classical: The Similarities

Let’s now make the transition to quantum theory. Here, I focus on the similarities between the classical example and quantum theory. In Section 6, I will discuss the differences between the classical and the quantum case.

Rather than immersing ourselves in the details of these arguments straight away, however, I would like to briefly comment on what such a discussion, of the similarities and differences between quantum theory and a classical example, could possibly establish. Now, we might observe that insofar as the dynamical evolution of betting-states relative to B was a *result*, a natural approach would be to place the previous discussion in the context of existing “reconstruction programs,” and investigate which assumptions would have to be modified in order to obtain a *different* result than in the classical case (i.e., the *actual* Schrödinger equation). What this would mean, provided that some of the more promising reconstruction projects proceed in terms of imposing information-theoretic constraints [8,9,11,30,31,32] is that we would have to investigate in what sense quantum theory limits the amount of knowledge an observer could, in principle, obtain. And certainly, this would sharpen our sense for what is “quantum” about quantum mechanics. While there is a lot to say about the similarities between the classical example and various reconstruction programs, the focus here will not be to provide such a systematic comparison or analysis. The reason is because it is not clear what these reconstruction programs establish from the point of view of interpretation. Timpson has articulated this issue like this: “By assumption [of a reconstruction project], the world is such that the information-theoretic constraints are true, but this is too general and it says too little: it is consistent with a wide range of ways of understanding the quantum formalism.” [29] (p. 177) In other words, once we have reconstructed the formalism, we should expect to be able to annex any interpretation to the result that we see fit. For this reason, the discussion here will instead take the following form:

- The goal of the discussion of the similarities between the quantum formalism and the classical example will be to provide a suggestive reason for why the above theorem can indeed be viewed as the counterpart of unitary evolution in quantum theory. This will rely on a result due to Lisi [25], who has proposed a heuristic derivation of the Schrödinger equations from similar assumptions as the ones that were required to prove the result of Section 3. Of course, and this is the important point here, such a discussion could only be suggestive (establishing too close a resemblance between the classical and the quantum case could only mean that we have made a mistake—the two cases are, after all, fundamentally different).
- The discussion of the *differences* between the two cases will be less suggestive. The argument will be structured around the thesis that *unlike* in the classical case, quantum theory is inconsistent with the assumption that each measurement has a determinate outcome. This argument will rely on a recent no-go theorem due to Frauchiger and Renner. [26], cf. [33] Since this no-go theorem is derived on the basis of the quantum formalism *itself*, this purports to show in what sense the formalism restricts the set of viable interpretations (this would not be visible if we stayed at the level of the reconstruction programs, for the reason articulated by Timpson).

Let me, then, begin the discussion of the similarities between the classical and the quantum case by briefly rehearsing the kinematical structure of the theory. Quantum mechanics is set in separable Hilbert spaces H. *Observables* are defined to be the self-adjoint elements of B(H), the set of bounded linear operators on the Hilbert space; the space of these observables is a von Neumann algebra: an involutive Banach-algebra that’s closed in the strong topology. *States* are defined as linear trace-class operators ℑ(H) on the Hilbert space (of trace 1). Notice that the state-space, too, is a vector-space: abstractly speaking, the state space is the *dual vector space* of the space of observables (playing such similar mathematical roles of being *structure-preserving functionals* makes for the analogy between number-state functions and quantum states). A special class of states, the so-called *pure states*, stand in one-to-one correspondence to the 1-dimensional subspaces of the Hilbert space (the so-called *rays*). Thus, we may (if we are dealing with pure states) use vectors in that subspace to represent the state (by convention, we take a vector of unit length although, strictly speaking, that still leaves a phase-ambiguity). To get probabilistic predictions from the theory, we rely on the Born rule, P(A|ρ)=Tr(Aρ) (where A is an observable and ρ is a density operator). This specifies *probabilities* (if A is a projection operator) and *expectation values* (for general self-adjoint operators). If ψ is a pure state, the expectation value is given by a vector-space product of the form (ψ,Aψ).

This mathematical structure, oversimplifying rather drastically, allows us to make probabilistic predictions for the outcomes of measurements, and hence it is sufficient to check quantum theory in experimental practice. But suppose that we aren’t satisfied with making only such probabilistic predictions, and that we want some form of understanding for how the experimental results are brought about. Suppose, in other words, we want to be realists about the claim that “System S is in state ψ.” How should we interpret this assertion?

On a first proposal, we might suggest that the state *represents* something in the world, in the sense that the world should be thought to be “isomorphic” (“structurally equivalent in the relevant ways”) to ψ. While this may sound tempting, there is something strange going on here from a conceptual point of view. Ordinarily, we would suppose that *systems* exist but not that states exist—and what type of *thing* or *object* in the world could possibly be isomorphic to ψ? As, e.g., Halvorson [34] points out, if we confuse *states* and the *objects of which they are states*, we run into the problem that “if states are objects, then states themselves can be in states. But then, to be consistent, we should reify the states of those states, and these new states will have their own states, ad infinitum. In short, if you run roughshod over the grammatical rules governing the word “state”, then you can expect some strange results.” [34] (p. 6) Consider, as a response, a second realist strategy: the suggestion that states play the functional role within the theory of assigning to the system a rather specific property: *“being in state* ψ.” This leads to what Halvorson calls a “state-to-property” link (he credits Wallace and Timpson [35] for this proposal). According to this, states no longer represent *objects*, but they represent a *property* of this object: *a system is said to be in state* ψ*, just in case it has the property of being in state* ψ. [34] (p. 24) However, it is not clear what exactly this implies with respect to ontology. As Halvorson remarks, “I suppose this claim is true. But I didn’t need to learn any physics to draw that conclusion. This is nothing more than a disquotational theory of truth.” [34] (p. 25) But the state-to-property link can be broadened: what certainly goes beyond the disquotational theory of truth, is the functional role states could play in assigning values to *other* observables. The set B(H) contains a whole fauna of self-adjoint operators, which represent physical properties. Relying on the eigenstate-eigenvalue link, a realist interpretation of the quantum state will, on this proposal, amount to the claim that states appropriately “track” the values (possibly unsharp!) that observables take for a given system. There is a host of technical complications connected to this proposal (most notably, the Kochen-Specker no-go theorem [36]). Here, however, I would only like to focus on the problem that has played center-stage in the discussion of realist interpretations of the quantum state: *the quantum measurement problem*.

To illustrate what the measurement problem consists in, we need to include the dynamical structure of quantum theory into our discussion. The first dynamical postulate of quantum theory is the *Schrödinger equation*, according to which states evolve unitarily. But Schrödinger’s famous cat thought-experiment [37] illustrates that this cannot be: macroscopic systems may evolve unitarily into superpositions that aren’t observed in experiment. In textbook presentations of the theory, one therefore typically encounters a further dynamical postulate: von Neumann’s *collapse postulate*, which tells us that when the measurement happens, the state after this measurement is represented by the projection operator corresponding to the eigenspace in which the system was observed to be. But this also cannot be: even leaving aside quarrels about non-locality, if the state represents some objective feature of the world, then collapse is a real physical process, which should have been brought about for a certain reason. Since quantum theory itself doesn’t provide such a reason, the collapse postulate, as Brown put it in the quote from the introduction, appears “mysterious.” [23] (p. 17).

Anti-realist interpretations escape this dilemma by dropping the assumption of realism about the quantum state. This, of course, raises the question of what role the quantum state is then going to play? The purported insight of, in particular, *QBism* [2,3,4,5,6,7], *neo-Copenhagen views* [8,9,10], and the *relational interpretation* [11,12,13] is that the state does not represent an objective feature of the world, but should be regarded as a codification of observer-relative information. It is a mere *consequence* of this suggestion that different observers may have to update their opinions in different ways (i.e., subjected to different constraints). Hence, these approaches, at least *prima facie*, are less threatened by the dualism of dynamical evolutions—but only insofar as they can make sense of both types of evolution as forms of rational opinion updating.

It would be too big a task to focus on all these interpretations here. Instead, I would like to highlight how this purported insight—the democratization of state ascriptions across observers—plays out for the case of Rovelli’s relational view, and how this insight bears on the measurement problem.

To this end, I will briefly outline the main commitments of the relational view. Now, on one way of reading Rovelli, his view is built around the following postulates:

- (*Equivalence of physical systems*) “All systems are equivalent: Nothing distinguishes a priori macroscopic systems from quantum systems.” [11] (p. 4).
- (*Relative facts postulate*) Any system has a quantum state relative to other physical systems (which we, depending on context, consider as the “observing” or “reference” systems).

To avoid any potential confusion, it is important to note that the (*Relative facts postulate*) is only compatible with *some* of Rovelli’s remarks, most prominently with his suggestion that “The core idea is to read the theory [of quantum mechanics] as a theoretical account of the way distinct physical systems *affect each other* when they interact (and not the way physical systems ‘are’).” [11] (*Relative facts postulate*) is inconsistent, however, with many other remarks of his, namely when he asserts what might be more aptly called the (*Empiricist facts postulate*):

- (*Empiricist facts postulate*) “A quantum description of the state of a system S exists only if some system O (considered as an observer) is actually “describing” S, or, more precisely, has interacted with S.” [11] (p. 6).

Clearly, the (*Relative facts postulate*) and the (*Empiricist facts postulate*) express different propositions. I will further restrain attention to the version of the relational view that is based on the (*Relative facts postulate*) (although most of Rovelli’s own ideas seem to rely on a commitment to the (*Empiricist facts postulate*)). There are several reasons for this (some of which will surface in Section 6). For the purposes of the current discussion, I would only like to note that the (*Relative facts postulate*) seems the more natural choice if we indeed take the classical example from Section 2, Section 3 and Section 4 as our intuition pump. The pertinent point is that the betting-states should be said to exist, relative to the different observers A and B, even if these observers don’t know what these betting-states are (as would be suggested by the (*Empiricist facts postulate*)). B’s best guess exists—in the same thin sense of “existence” in which any mathematical entity can be said to exist—even if B doesn’t follow the calculation from Section 3. Similarly, the betting-state for A after the next game is either 〈w+1, d, l〉 or 〈w, d+1, l〉 or 〈w, d, l+1〉. Even though we don’t know *which* one it will be, we are sure *that* one of these is correct. Hence, there should be no requirement that either A and B know what the correct betting-state is (relative to either of them), in order to say *that* there is such a state.

To illustrate the workings of the (*Relative facts postulate*), think about how mathematics is used in physics more broadly. Take, for instance, a classical particle that is dropped from a high altitude, while being subjected to air resistance. The particle’s trajectory will, in certain models, be the solution to a (non-analytically solvable) differential equation of motion. Now, to say that this mathematical description is true because the interactions that occur (between the gravitational field, the particle, and the air) are nature’s way of “solving this equation” is obviously nonsense (how could nature solve the equation, if it is not analytically solvable?). The ones solving the equations are certainly going to be the users of the theory. To say that this equation is “true” says something about the *solution* of the equation, but not anything about the *process* by which this solution is obtained—although the equation tells us something about the interaction, the interaction itself is irrelevant for what the equation says. The same applies to probability assignments on the relational approach that is based on the (*Relative facts postulate*). The *process* of calculating or acquiring information about relative states is as irrelevant, for the claim that there *are* such relative states, as the process of finding solutions to the equations of motion is for the claim that the equations of motion are true. Hence, the relational view that is based on the (*Relative facts postulate*) manages to incorporate the key conceptual point (that states are always relative to a reference system) in a way that is consistent with how mathematical models are applied in physics more broadly. Secondly, it is also worth emphasizing that assuming there to be facts about states relative to physical reference systems is not to say that unanimated objects use probability ascriptions to inform their betting behavior (just like nature doesn’t solve differential equations). Of course, the state ascriptions are made, remembered, and exploited, by the users of the theory for their epistemic or decision-theoretic purposes (just as in the case of the particle being dropped, the equations are solved, and exploited, by agents for their epistemic or decision-theoretic purposes). The claim is, rather, that nature just ends up behaving in a way that is compatible with what would have been predicted if anyone had actually ascribed, recorded, or calculated these states (just like nature ends up behaving in a way that solves the equation of motion, independently of whether anyone actually *solves* the equation, or has any determinate record of the initial conditions). Only conscious agents rely on ascribing relative states, but consciousness itself is irrelevant for the claim that there are relative states. These remarks are intended to show that the relational view manages to deflate the subject/object dichotomy of QBism or neo-Copenhagen approaches (since the observers, on the relational approach, are thought of as purely physical systems). One requirement we certainly must place on the reference systems, however, is that they are the kinds of things that can *in principle* acquire information about a system, by virtue of *interacting* with it in the appropriate way (hence the (*Relative facts postulate*) requires the reference systems to be *physical* systems).

Departing now from these assumptions, let’s return to the main thread that was left hanging above: how does the democratization of quantum state ascriptions, in the way that is prescribed by the (*Relative facts postulate*), bear on the quantum measurement problem? What is suggested by the remarks so far, is that the problem of understanding dynamical evolution in relational quantum mechanics will be solved by the modelling our answer on the question: *How should observers update their relative state ascriptions in the “best” way*? Let me reemphasize that it is a mere consequence of this view that we may be forced to admit that, just as in the classical case, different observers might have to update their opinions in different ways. If this would turn out to be the case, this would underscore the (*Relative facts postulate*).

Let’s use the example of the Wigner’s friend thought-experiment [28] to illustrate how this line of thought will be developed (the presentation here follows Rovelli in Ref. [11] (pp. 2–4)). Suppose that there are two physical reference systems—referred to as “observers”—O and P, as well as a further system S. So, according to the relational view, S has a state relative to both O and P. The experimental protocol is such that O is going to conduct a measurement on S, but P doesn’t participate in the process. For concreteness, let S be a spin-half system, and let the measurement that O performs on S be a spin measurement along the z-axis. The relative states *after* the preparation procedure but *before* O’s measurement are given by:

ψ(S, O)=α |up〉 + β|down 〉 

ψ(S+O, P)=α|up ⨂ ″ready″+〉 β|down ⨂ ″ready″〉

Here the obvious notational convention is that ψ(S+O, P) denotes the state of the joint system S+O relative to P (the second term after the tensor product denotes the state of O). Note that because ψ(S+O, P) is a product state O and P will initially agree on the relative state of the system S (“tracing out” the observer O from ψ(S+O, P) yields the state ψ(S, P) which is the same as ψ(S, O)).

Next, O carries out her measurement on S in the spin-z basis. After this measurement, the state of S relative to O will collapse, according to the *collapse postulate*, into one of the eigenstates “up” or “down.” As was mentioned in the introduction, this can be justified by the proponent of the idea that the quantum state is observer-dependent by appealing to Lüder’s rule [18]: oversimplifying slightly, the projected state agrees, in its Born probability assignments to observables, with the canonical generalization of the notion of conditional probability (conditioned on the observed outcome) to a quantum mechanical setting (in which the objects, to which probabilities are assigned, are elements of an ortho-modular lattice rather than a Boolean algebra). Therefore, *relative to O*, who has access to the measurement *outcome*, it is rational to use, as the basis for future predictions, the collapsed state [20] (pp. 171–175), [21], [22] (pp. 170–173).

If P, however, doesn’t interact with S+O, then the standard quantum mechanical prediction for the state of S+O relative to P *after* O’s measurement will be the unitarily evolved state:

ψ(S+O, P)=α|up ⨂ ″up″+ β|down ⨂ ″down″

But now the question arises: can the proponents of observer-relative interpretations of the state justify unitary evolution as a form rational probability updating? By now it will be apparent that the suggestion is to view the theorem for optimal rational opinion change we have met in Section 3 as the classical counterpart of unitary evolution in quantum mechanics, in just the same way in which Bayes’ theorem may be viewed as the classical counterpart of the projection postulate in quantum mechanics.

To substantiate this claim, I will use a result by Lisi [25], according to which P’s question is presented in an identical form as the problem of optimized probability updating that was discussed in Section 3. Lisi, who explicitly puts his approach in the context of Rovelli’s relational interpretation (cf. [38]), presents his argument in the language of the path-integral formalism developed by Feynman. [39] In this formalism, we consider all possible paths of a system between two fixed points, each of which is described by a configuration q(t). We then ascribe a probability p[q] to each path. Then, it can be shown that solving the following optimization problem is equivalent to the path integral formulation of quantum mechanics:

- *Optimization problem for the external observer in quantum mechanics*. We require that the entropy—H=−∫Dq p[q] log(p[q])- is minimal, subject to the constraints that (1) the probabilities associated with each path sum up to 1: ∫Dq p[q]=1 (normalization), and (2) the expectation value of the action functional is fixed to be S=∫Dq p[q] S[q].

Solving this problem (using the method of Lagrange multipliers), Lisi derives the form of the wavefunction ($\phi =e^{-\mathrm{iS}/\hbar }$). From this, the Schrödinger equation, i.e., unitary time-evolution, follows in the standard manner. By virtue of requiring the expectation value of the action functional to be constant, Lisi refers to his “heuristic derivation” [25] (p. 1) as an attempt to reconstruct quantum theory from a universal action reservoir. [25] (p. 2) Despite acknowledging that his derivation is at best heuristic, he argues that “The main product of the work is the proposal of a new physical principle for the foundation and interpretation of quantum mechanics: a universal background action.” [25] (p. 4).

Notice that this situation is completely analogous to the one we encountered in Section 2 and Section 3! There are two observers (the *participating observers* A and O vs. the *non-participating observers* B and P), with access to different pieces of information (A and O know the *outcome* of the interaction, while B and P only know *that* the interaction has taken place). These differently situated observers are expected to update their information subject to different sets of conditions of optimality (relative to their respective epistemic situations). As before, the non-participating observer wishes to maximize entropy; and in both cases, quantum and classical, we end up with a dualism of dynamical evolutions.

From these assumptions and results, Rovelli’s *main observation* follows:

“**Main observation:** In quantum mechanics different observers may give different accounts of the same sequence of events.”

[11] (p. 4)

To summarize, the reason why they must do so is because: (1) of the presupposition that the relative states do not correspond to, or represent, real states of affairs, but should be understood as “encoding observer-dependent information about the system,” and (2) different observers should update their information relative to different criteria of optimality and rationality.

The (*Relative facts postulate*) therefore provides a *natural* explanation for why there is more than one possibility to update one’s opinion: different observers, depending on whether they know *the outcome* of an interaction (O’s knowledge) or only *that* the interaction between O+S has taken place (P’s knowledge), might have to update their beliefs in different ways in order to act the most rationally. In that sense, the suggestion that the quantum state is a codification of observer-relative information offers an elegant (dis-)solution to the measurement problem.

What we have seen so far, then, is that there is a suggestive and far-reaching analogy between our classical example and quantum mechanics. The proponents of the view that states are observer-relative, therefore, have the means to justify the view that quantum theory is a new form of (non-Boolean) probability theory. However, it is not immediately obvious what the democratization of quantum state ascriptions—as postulated by in the (*Relative facts postulate*)—implies from the point of view of interpretation. On the one hand, there clearly is no *logical* requirement that any such story of observer-dependent facts must be completed by some realist-type ontological story. But, of course, we might still *want* to find such a story. At least ideally, a stout non-representationalist should also provide an argument to the effect (not only that one *needn’t* give a realist story but) that one *couldn’t* give such a story. In Section 6, I will outline what I consider the strongest argument to establish precisely this conclusion.

## 6. Quantum vs. Classical: The Differences

As always, understanding an analogy, to a great extent, consists in the understanding of how it breaks down. While I have, so far, focused on the similarities between quantum and classical probability models, I will now discuss their differences.

The cue for understanding how quantum and classical probabilities differ, derives from the observation that in the *classical case*, it is natural to argue that A’s betting-state is *privileged* over B’s. After all, A has, unlike B, *actually* observed the outcome of the game, while B’s state is only a “best guess.” In the classical case, then, not all models are *epistemically equal*, in the sense that we might say that “A knows *more* than B.” This subsequently allows us to assert that A’s descriptions, i.e., the betting-states relative to A, *corresponds to something in reality*, while B’s description has a merely epistemic function. For this reason, we may, in the classical case, envelop the probabilistic model in a straightforward ontological story of what the world is like.

So, we must investigate whether this situation also obtains in quantum mechanics: can either of the two perspectives (O’s or P’s) be privileged over the other? This, certainly, would undermine the (*Relative facts postulate*) from Section 5, which only has real bite if we regard both O’s and P’s relative state ascriptions as *equally valid* codifications of what is rational to believe from their respective perspectives. Section 6.1 argues why these privileging strategies are unsuccessful. Oversimplifying the matter slightly, the crux of the issue will be that *unlike* in the classical case, measurement outcomes, in quantum theory, can’t (in general) be said to be *determinate*. This, after all, is the basis on which (in the classical case) epistemic privilege is handed out to the participating observer A. To establish that quantum mechanics differs from classical theories in precisely this respect, I will rely on a recent thought-experiment by Frauchiger and Renner [16] (many thanks to Richard Healey for bringing this to my attention).

But even so, there is still a third possibility that we will need to consider, namely that neither description is privileged, but that they are compatible with some underlying realism (a situation that obtains, e.g., for *velocity ascriptions* in special relativity).

I will try to illustrate how one might come to believe that *neither* of these three options—(1) O’s state is privileged, (2) P’s state is privileged, (3) O’s and P’s states are compatible with some underlying ontology—obtains. If the overall argument is indeed deemed successful, we will have a strong rationale for claiming that quantum theory can’t, in general, be supplemented with a realist story (the argument here may be thought of as an elaboration and development of Rovelli’s discussion of these issues in Ref. [11], in light of more recent technical results).

### 6.1. Privileging Strategies

Section 6.1.1 will discuss the suggestion that O’s state is privileged. Section 6.1.2 will discuss the possibility that P’s state is privileged. Both will be rejected.

#### 6.1.1. Treating O’s State as Privileged

The first stab we might take at privileging one observer would be to follow the classical analogy. According to this, we might argue that in our Wigner’s friend example, only observer O’s description (i.e., the collapsed state) is a true description of reality, while P’s state has only an *epistemic*, but no directly *representational* function (i.e., that of quantifying P’s degree of ignorance with respect to the true state, which is correctly represented by ψ(S, O) after collapse has occurred). To motivate this proposal, one could argue, first, that there is an important asymmetry in the Wigner’s friend example: O has, unlike P, *actually* received a measurement outcome, which mirrors our reasoning in the classical case (in which A’s description was privileged). The problem with this proposal is that P can use her state to make predictions for future measurement outcomes, and for all we know, these predictions are empirically adequate. Therefore, the experimental facts undermine this strategy. [11] (p. 5) Since I don’t know of any view that rejects this argument, I feel confident to move on to the next possibility. (The situation is more complex if either of the systems O or S is macroscopic. In this case, interference effects are seldom observed. However, rather than being forced to assume that the ascription of a superposition is *false*, P could rely on decoherence-theory to at least explain why no interference effects are observed.).

#### 6.1.2. Treating P’s State as Privileged

In a radical change of heart, we might abandon the idea that O’s state is privileged and move towards the other extreme of proposing that P’s state is privileged. On such a view, collapse never occurs: all there is, is the unitarily evolving state, as prescribed by the Schrödinger equation. Of course, this also requires an argument. In this section, I will consider—and reject—two possibilities.

##### 6.1.2.1. Argument from Interference

The argument that’s sometimes favored in the literature picks up the thread from Section 6.1.1. Using the unitarily evolved state, P can predict interference effects that can be confirmed experimentally, but that cannot be explained on the basis of the collapsed state. But while P’s unitarily evolved state has an important role in guaranteeing the empirical adequacy of the theory, this may provide just enough reason not to “*under*”privilege P’s state. It doesn’t follow that we can “*over*”privilege this state.

One way to see this, is by noting that the argument from interference to reality is not logically valid. A counterargument can be constructed within the ontological models framework that was briefly introduced in Section 2.2.3. It can be shown that, e.g., Spekkens’ toy-model, whose states must unambiguously be interpreted as epistemic, can reproduce interference effects, at least under certain constraints. [27] (pp. 3, 11) This provides the counterexample, which establishes that the inference from interference to reality is not logically valid (in Spekkens’ words: “All this argument demonstrates… is a lack of imagination concerning the interpretation of coherent superposition within an epistemic view” [27] (p. 11); cf. Leifer in Ref. [15] (pp. 78–79) for a more elaborate version of the argument).

##### 6.1.2.2. Argument from Scientific Realism: Explanatory Virtues

One might respond to this by pointing out that the utility of the ontological models framework for foundational issues is controversial. Instead of cosmetically altering the formalism (for example, by adding an extra layer of ontological models) we should take the mathematical structures provided by the formalism itself seriously—i.e., *literally*. In that sense, the argument for privileging P’s state could derive from the scientific realism debate.

This line of thought can be developed as follows. First, observe that insofar as the collapse postulate (as Brown [23] (p. 17) had put it) appears mysterious, we might reject it as an *ad hoc* modification of the theory, and this is unacceptable from the point of view of a realist interpretation of science. Furthermore, although the argument from interference to reality is not logically valid, it might still be the case that such an inference is *plausible*; after all, one might reason, if the collapse postulate is not “really” part of the quantum formalism, then the fact that P’s state expresses some actual states of affairs is the best explanation for why quantum theory is so empirically successful. Hence, observer O’s collapsed state should be underprivileged, and observer P’s unitarily evolved state should be taken as the true description of the world.

This line of reasoning is endorsed by proponents of Everett’s many-worlds interpretation [40,41], which has been argued to be the only strategy that reifies scientific realism and quantum theory [41] (pp. 35–39). In this section, I will outline how the case against this line of reasoning might unfold (thus, I use Everett’s interpretation as a paradigmatic example of a realist interpretation, to illustrate what problems may arise in the context of such interpretations).

Let me, first, expand on the core idea behind the Everett interpretation. To fix the attention, let’s return to the Wigner’s friend example. According to Everettian views, the final state of the entire multiverse, before P’s interaction with S+O but after O’s interaction with S will be given by:

ψ(S+O+P)=α|up ⨂ up ⨂ ready〉+ β|down ⨂ down⨂ ready〉

Proponents of the Everett interpretation then argue that after O’s measurement, the world has split into two distinct branches, which are to be referred to as “worlds”—one in which O has received outcome *up* and one in which O’s trans-worldly counterpart has received outcome *down*. According to Wallace’s [41] influential construal of the Everett interpretation, the language of “worlds” is justified because two further conditions are satisfied:

- (*Non-interference*) The different terms in the superposition are “causally shielded” from one another. They are eigenvectors of some observable (thus they are necessarily orthogonal), and hence no interference effects can be observed if we measure in that basis. [41] (pp. 60–63).
- (*Functional instantiation of properties*) Each of the non-interfering terms of the superposition represent (= is functionally/structurally equivalent to) a world that consists of objects which instantiate (at least some) determinate properties (such as a particle having the determinate properties of being either “spin-up” or “spin down”). (*ibid*.)

The merits of this proposal are quite attractive. By equipping the formalism with a straightforwardly realist interpretation (in terms of real physical objects that instantiate at least some determinate properties) we gain the best possible *understanding* of what the world is *really like*, if quantum mechanics were *literally* true. Since these explanatory benefits are only accessible to the realist, the argument in favor of the reality of P’s state derives from the scientific realism debate.

Of course, many-worlds interpretations are not free of problems. The two problems most widely recognized in the literature are the so-called *preferred basis problem* and the *probability problem*. Both have been discussed extensively in the literature and there is no need to repeat these arguments here. [41,42,43,44,45] Instead, I would like to very briefly mention one problem that can be generated due to the recent no-go result by Frauchiger and Renner. [26]

We begin by observing that the many-worlds approach still needs to make sense of the usage of the Born rule in experimental practice. One prominent strategy proceeds by giving the Born rule an *epistemic function* [41,43,44,45], namely in the following sense. A proponent of the many-worlds approach believes that only unitary evolution tracks real changes in the world. Thus, in our Wigner’s friend example, there are (after O’s measurement on S) *two* copies of P. The state of the multiverse ψ(S+O+P) represents two worlds, each of which contains a still uncertain observer P in the “ready” state. In the case in which P happens to be a conscious agent, therefore, a question arises for P: which of the two versions of herself is she? This is referred to as “self-locating uncertainty” that arises in the many-worlds interpretation. The proposal then is that the Born rule provides the quantitative measure for P’s degree of uncertainty: P can use this quantitative measure for her decision-theoretic purposes. [41,43,44,45].

Notice, however, that P’s question only makes sense relative to the assumption that each version of O has received a definite outcome during her measurement on S. Here is why: there is a trivial sense in which it is silly to ask “Where am I?” In that trivial sense, the answer is always: “I am *here*…in *my* world!” But there is sense in which this question is not trivial, namely if it is understood are referring to external circumstances: “Which of the different physically possible worlds is *my* world?” And this only makes sense if we have an account of what these different worlds are like. Only if there really are distinguishable worlds (in terms of their properties such as: there is one world in which O has received outcome “up”, and one in which O has received “down”) does it make sense for either version of P to ask “In which of the two worlds am I?” *Prima facie*, this doesn’t sound like a problem; after all, this was precisely the reason to introduce (*Functional instantiation of properties*) in the first place (i.e., to justify talk about “worlds” by virtue of the idea that the different terms in the superposition could be said to represent objects that instantiate at least some determinate properties).

But now we phrase an attack against this as follows. Due to a recent no-go theorem by Frauchiger & Renner [26], who develop on Hardy’s paradox [46,47], there are good reasons to believe that quantum mechanics is inconsistent with the assumption that, in general, each term in a superposition can be said to represent a world with determinate properties. The authors discuss an extended version of the Wigner’s friend situation. In this extended version, there are *two* “friends” and two “external observers.” They then set out to prove that no theory that is empirically equivalent to quantum mechanics can be both (1) self-consistent and (2) be committed to the claim that each measurement has a unique, determinate outcome. The details of their argument would distract from the current discussion (Appendix C gives sufficiently elaborate version that contains the relevant details; for criticisms of their argument, see [48,49,50]). The conceptual point is that this result provides a reason to believe that the Everettian commitment to (*Functional instantiation of properties*) might be inconsistent with quantum mechanics, at least in certain experimental set-ups. (In the preprint, Frauchiger and Renner suggested that their argument lends support to the Everettian view. In the published version, however, they have expressed doubts about whether the Everett interpretation is consistent with their no-go theorem, at least if branching is supposed to be objective. [26] (p. 10) The argument here (and in Appendix C) should strengthen these doubts. Cf. [33] (p. 7)).

From the point of view of interpretation, the consequences are twofold. For one thing, this shows that P cannot, in general, discriminate between the two worlds (because she cannot tell us what the different worlds are like). Therefore, P also cannot meaningfully ask questions about self-locating uncertainty (she can’t question in which of the branches she has ended up in, if she can’t tell us what these branches are like). Hence, we might ask the Everettian: if (*Functional instantiation of properties*) is indeed inconsistent with quantum mechanics, then, on a decision-theoretic interpretation of the Born rule, this rule aids users to make decisions... *about what?!* But clearly, this doesn’t only challenge the decision-theoretic interpretation of the Born rule. It also undercuts the Everettian strategy in a deeper sense: *if* the motivation for the many-worlds interpretation was that it provides us with an *understanding*—in the peculiarly realist sense of providing an account of *what the world would have to be like if the theory were literally true*—that motivation has now been lost! And with it the argument we are currently considering—that P’s state should be regarded as epistemically privileged *because* it provides us with such an understanding—disappears as well.

This concludes the discussion of the potential privileging strategies. If all these strategies are unsuccessful, the two descriptions—i.e., the states relative to O and P—should be seen as *equal*. This breaks the analogy to the classical case.

### 6.2. (Hidden) Commonalities?

There is one last case that we still need to consider: what if neither of the two states—the one relative to O and the one relative to P—are privileged, but that they are not actually *different* descriptions of what the world is like, in the sense that there is, really, some underlying commonality? In this most general way of putting the problem, this question is unanswerable. But we can identify two important aspects, which will be discussed in turn.

#### 6.2.1. The Quantum State Is Not Epistemic!

The first brings us back to the ontological models framework that was introduced in Section 2.2.3: what if two different quantum states—i.e., those relative to O and P—are interpreted as assigning a non-zero probability to some underlying ontic state (from an ontic state-space that might be part of some hidden-variable framework)? Couldn’t it be the case that two different quantum states each assign a non-zero probability to one and the same ontic state? On such a scenario, the two descriptions would certainly be compatible with a realist account, although neither description would be a “direct” representation of what the world is like. This possibility, however, is ruled out by the PBR theorem [14]—in the ontological models framework, each ontic state is compatible with only one quantum state! Thus, different quantum state ascriptions, if both are valid, are incompatible with the assumption that the world is in some determinate state. At the same time, Section 2.2.3 already illustrated how one may consistently argue that a state is “ontic” (in this peculiar sense of being “non-epistemic”) while shying away from attributing any representational function to such a state. (There is an important comment to be made here about the relationship between this argument and the distinction between different versions of relational quantum mechanics, which are based on what Section 5 referred to as the (*Relative facts postulate*) and the (*Empiricist facts postulate*). On the latter, though perhaps not on the former, the possibility of ascribing a state to oneself is ruled out [11] (p. 15). Thus, there is a trivial sense in which, on the empiricist version, O’s and P’s states are not compatible: both O’s and P’s states are about different systems. Once we move to the version of relational quantum mechanics based on the (*Relative facts postulate*), state ascriptions to oneself may become possible. Thus, it is *this* version of the relational view that potentially needs to exploit the PBR theorem to establish that relational quantum states are incompatible with the assumption of underlying realism.).

#### 6.2.2. Measurement Outcomes Are Not Objective!

There is yet another sense in which the two state ascriptions, if neither is privileged, may be compatible with an ontology, namely an ontology of *measurement outcomes*. After all, even though we may have rejected a realist account of how measurement outcomes are brought about, we might still believe in the existence of these outcomes. Such a view is indeed part and parcel of Rovelli’s own thinking about these matters. Although his view is built around the idea that relative state ascriptions vary with the reference systems, he nevertheless maintains that quantum theory is a theory *about* (in an ontological sense) measurement outcomes: “in [relational quantum mechanics], physical reality is taken to be formed by the individual quantum events (facts) through which interacting systems (objects) affect one another. Quantum events are therefore assumed to exist only in interactions and (this is the central point) the character of each quantum event is only relative to the system involved in the interaction.” [51] (p. 2).

This claim is grounded in the alleged objectivity of measurement outcomes, which relates to Deutsch’s influential discussion of the Wigner’s friend thought experiment. [52] In the simple Wigner’s friend example, one can show that although the *character* of the event is observer-relative (as O and P ascribe different relative states), the fact *that* they occurred is not [52], [51] (pp. 7–9). Hence, measurement outcomes can, in the Wigner’s friend example, be thought of as objective, i.e., *observer-independent*. The problem with this, however, is that the technical aspects on which these ideas are founded are artefacts of certain specific cases, like the simple Wigner’s friend example discussed above. In a more general context—like in the extended Wigner’s friend experiment discussed by Frauchiger and Renner [26]—this conclusion is no longer true (again, the reader might wish to consult Appendix C). Therefore, even the thin ontology of measurement outcomes, can’t consistently be postulated (at least not in general) as long as quantum theory remains our most successful empirical theory.

Finally, we have exhausted the space of possibilities: the above argument illustrates why the (classically expected) dualism of dynamical evolutions in quantum theory can’t be ramified with our classical intuitions. *Unlike* in the classical case, in quantum mechanics the different descriptions, offered by different observers O and P, can’t be endowed with a realist interpretation.

## 7. Conclusions: Representation Lost

The conclusion of this paper did not follow from any philosophical ideology. On the contrary, it was based on a conceptual analysis of technical results—both in formal epistemology, but also in quantum foundational research. This argument had three interdependent components: (1) Different observers will, because of the different pieces of information available to them, ascribe different quantum states to one and the same system. (2) In general, we cannot supplement the observer-relative quantum states with some kind of mechanical/substance-type story of what the world is like. (3) To make these radical-sounding claims more easily digestible, I presented a simple example, which also instantiated what I believe to be the most promising strategy to make sense of quantum theory. The toy-example, specifically, illustrates that operating within a framework of non-representational states is simply an artefact of certain modelling practices (and does not entail commitment to either skepticism or solipsism).

Of course, the usage of non-scientific examples or toy-models for foundational issues might be contested. Let me therefore conclude with some brief remarks about what I take to be the importance of such simple-minded examples as the one that was discussed above. The reason why it is hard to make sense of quantum theory is because the theory invites a battle of intuitions: between what science could not *possibly* be doing, but nevertheless *seems* to be doing… In our strive for clarity, there is only so much we can do. We might, for one, inquire into the mathematical architecture of the theory (by arriving, for example, at such rightly celebrated results as the PBR theorem). But we can also aim to provide examples and counterexamples, which allows us to better separate what is truly *necessary* from what is merely *plausible*. The football example aims to do just this. Even if it isn’t anything more than a crutch for our intuitions, it illustrates where these intuitions come from. And, at the very least, such examples can provide a proof of concept that a certain set of beliefs can consistently be upheld, even in light of strong results or intuitions that might suggest the opposite.

## Figures and Tables

**Figure A1:**
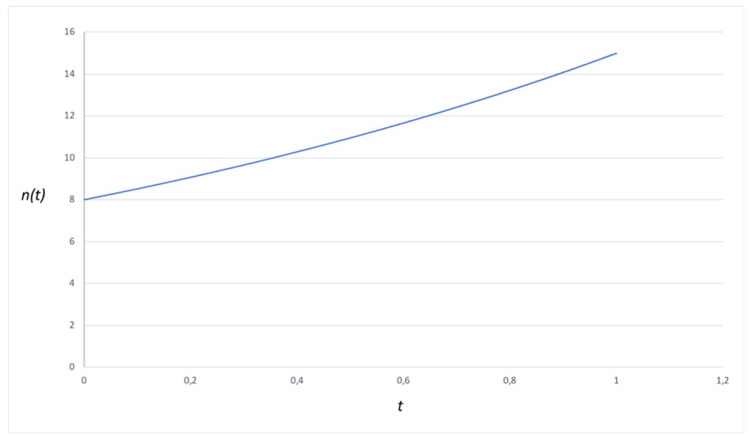
Total number of games as a function of time.

## Appendix A. Proofs of the Theorems from Section 3

I will provide a sketch of the proofs for the three theorems that were mentioned in Section 3. For a complete discussion of the proofs, see Hughes and van Fraassen in Ref. [24], and van Fraassen in Ref. [20] (pp. 68–73).

In the following, let $\bar{x}=\langle x_{1},\ldots ,x_{n}\rangle$ denote a betting-state.

**Theorem.** 
*A transformation U is a symmetry if and only if there exist positive real numbers* $u_{1},\;\ldots ,\;u_{n}$ *such that* $U(\bar{x})\langle =u_{1}\;x_{1},\;\ldots ,u_{n}\;x_{n}\rangle$ *. [24] (p. 857), [20] (pp. 71–73).*

**Proof.** If there exist positive real numbers $u_{1},\;\ldots ,{\;u}_{n}$ such that for all $\bar{x}$, $U\left(\bar{x}\right)=\langle u_{1}{\;x}_{1},\;\ldots ,u_{n}{\;x}_{n}\rangle$, then clearly U is a symmetry. To prove the converse, take the unit betting-state $\bar{1}$, whose components are all equal to 1. Let $U\left(\bar{1}\right)=\langle u_{1},\;\ldots ,u_{n}\rangle$. Since U is a symmetry: $U\left(\bar{x}\right)/U\left(\bar{1}\right)=\bar{x}/\bar{1}=\bar{x}$. Let $U\left(\bar{x}\right)=\bar{x}\prime$. Then we may write: $\langle x_{1}^{\prime }/u_{1},\;\ldots ,x_{n}^{\prime }/u_{n}\rangle =\langle x_{1},\;\ldots ,x_{n}\rangle$. Since two betting-states are equal if and only if their components are equal, and since the components of betting-states must be positive real, the result follows. □

**Theorem.** *If two betting-states*$\bar{x}\left(0\right)$*and*$\bar{x}\left(t\right)$*are related via a uniform motion*$\bar{x}\left(t\right)=\;U\left(t\right)\left(\bar{x}\left(0\right)\right)=\langle u_{1}\left(t\right)\;x_{1}\left(0\right),\;\ldots ,u_{n}\left(t\right)\;x_{n}\left(0\right)\rangle$, *then there exist positive real numbers*$k_{1},\;\ldots ,\;k_{n}$*such that*$u_{i}\left(t\right)=\;e^{k_{i}t}$*([24] (p. 858), [20] (pp. 72–73)).*

**Proof.** From before, we know that $U\left(t\right)\left(\bar{x}\left(0\right)\right)=\langle u_{1}\left(t\right){\;x}_{1}\left(0\right),\;\ldots ,u_{n}\left(t\right){\;x}_{n}\left(0\right)\rangle$, where the $u_{i}$’s are positive real. Since U is a uniform motion, it follows that $u_{i}\left(t_{1}+t_{2}\right)=u_{i}\left(t_{1}\right)u_{i}\left(t_{2}\right)$. Hence, we may define a function $f_{i}\left(t\right)=\ln\left(u_{i}\left(t\right)\right)$, which is additive in t. From elementary calculus, we know that therefore $f_{i}\left(t\right)=k_{i}t+C$. Since $u_{i}\left(0\right)=1$, it follows that C=0, which proves the theorem. □

**Theorem.** *There exist constants v & w such that the*$u_{i}\left(t\right)$*’s are given by*$u_{i}\left(t\right)=\;e^{vt}\;e^{ws_{i}t}$.

**Proof.** We prove this by using the method of Lagrange multipliers. [24] (p. 861) The Lagrangian is given by: $L=\sum k_{i}x_{i}\left(t\right)-w\left(\sum s_{i}x_{i}\left(t\right)-r\left(t\right)\right)-v\left(\sum x_{i}\left(t\right)-n\left(t\right)\right)$. Here, w and v are Lagrange multipliers. When we derive this with respect to component $x_{i}$ and set the result equal to 0, we get: $k_{i}-{\mathrm{ws}}_{i}-v=0$. Hence the result follows. The two Lagrange multiplies are fixed by the conditions that (1) ($\sum {\;e}^{{\mathrm{ws}}_{i}t}x_{i}\left(0\right)){\;e}^{\mathrm{vt}}\;=n\left(t\right)$ and (2) ($\sum s_{i}{\;e}^{{\mathrm{ws}}_{i}t}x_{i}\left(0\right)){\;e}^{\mathrm{vt}}=r\left(t\right)\;$. □

## Appendix B. An Example for the Theorem in Section 3

In this appendix, I will try to illustrate why the theorem from Section 3 produces reasonable results. This investigation is spurred by the worry that since the components of the betting-states evolve exponentially, this implies (among other things) the rather counterintuitive result that the total number of games played n(t) evolves as a sum of exponential functions. To see why this result is less counterintuitive, and less threatening, than it might first appear to be, it will be best to just consider a concrete example.

So, let’s assume the following boundary conditions: (1) At t=0, the betting-state of a team relative to B is assumed to be 〈3, 3, 2〉. Hence, the team has played 8 games. If we are assuming the standard choice of number-state function s=〈3, 1, 0〉 this means that the team has collected 12 points at the initial time. (2) Let the boundary condition for the final time t=1 month be that (a) the team now has played 15 games in total, and (b) that it has picked up 22 points overall.

Using these boundary conditions, it is straightforward to calculate the values of the Lagrange multipliers to be: v≈0.66167 and w≈−0.02229. Relying now on the theorem from Section 3, the final betting-state relative to B will therefore be given as 〈5.44, 5.69, 3.88〉. Of course, it has to be admitted that this is not particularly useful—it is left open whether B should guess that the team is really in the betting-state 〈6, 4, 5〉 or in the betting-state 〈5, 7, 3〉 (which were the only possible betting-states to begin with; the remaining two betting-states that the final number-state ascription declares possible—〈7, 1, 7〉 and 〈4, 10, 1〉—are ruled out by the initial conditions, since the team cannot have fewer draws or losses than it had started out with).

However, to probe whether result is at least reasonable, we can use it to illustrate what the overall evolution of n(t)—the total number of games as a function of time—looks like. This function is just the sum of the components of the betting-state, i.e., it is a sum of exponentially evolving functions in time. n(t) is plotted in Figure A1 for 0<t<1:

Hence, the evolution of the total number of games, for the time-frame in question, is very close to being linear. This therefore illustrates that the counterintuitive result—that n(t) is a sum of exponential functions—will not be threatening in concrete examples. Therefore, the calculations from Section 3 produce reasonable results.

## Appendix C. The Frauchiger and Renner 2016 Thought Experiment

The thought-experiment constructed by Frauchiger and Renner [26] consists in an extended version of Wigner’s friend example. There are two friends (F1 & F2), Wigner (W) and his assistant (A). F1 prepares a *quantum coin* at t_0_ and depending on the outcome of her measurement (*heads* or *tails*) at t_1_, she sends a spin-1/2 particle to the second friend F2. This spin particle is going to be prepared in either the z+ or the x+ direction, depending on whether F1 has observed outcome *heads* or *tails* respectively. F2 then conducts a measurement in the *z*-basis at t_2_ on the particle she has received from F1. At t_3_ the assistant A conducts a measurement on F1 in the basis *ok/fail* (which is an equally weighted superposition of the *heads*/*tails* basis—with a minus and a plus sign respectively—on the Hilbert space of F1). At t_4_ Wigner conducts a measurement in the *ok/fail* basis on F2. The experiment is repeated many times, until Wigner and A have both received the outcome *ok* in their respective measurements on F2 and F1.

We then consider the final state of the entire system, which can be written in five equivalent ways, given the initial state of the quantum coin (unnecessary normalizing factors and factors in the tensor product have been omitted):

$$\left(A\right):\;\psi \;\sim \;({\mathrm{tails}}_{F1}⨂{\mathrm{up}}_{F2}+{\mathrm{tails}}_{F1}⨂{\mathrm{down}}_{F2}+{\mathrm{heads}}_{F1}⨂{\mathrm{down}}_{F2})$$

$$\left(B\right):\;\psi \;\sim \;(\sqrt{2}{\;\mathrm{fail}}_{F1}⨂{\mathrm{down}}_{F2}+{\mathrm{tails}}_{F1}⨂{\mathrm{up}}_{F2})$$

$$\left(C\right):\;\psi \;\sim \;({\mathrm{tails}}_{F1}⨂{\mathrm{fail}}_{A}+\frac{1}{\sqrt{2}}{\mathrm{heads}}_{F1}⨂\left({\mathrm{ok}}_{A}+{\mathrm{fail}}_{A}\right))$$

$$\left(D\right):\;\psi \;\sim \;(\left(\frac{1}{\sqrt{2}}-1\right){\mathrm{ok}}_{F1}⨂{\mathrm{ok}}_{A}+\left(\frac{1}{\sqrt{2}}+1\right){\mathrm{fail}}_{F1}⨂{\mathrm{fail}}_{A})$$

$$\left(E\right):\;\psi \;\sim \;(3{\;\mathrm{fail}}_{W}⨂{\mathrm{fail}}_{A}+{\;\mathrm{fail}}_{W}⨂{\mathrm{ok}}_{A}+{\;\mathrm{ok}}_{W}⨂{\mathrm{fail}}_{A}+{\;\mathrm{ok}}_{W}⨂{\mathrm{ok}}_{A})$$

From these results, we will now prove the claim from Section 6.1.2.2 that (*Functional instantiation of properties*) is inconsistent with quantum mechanics. From an Everettian standpoint, we assume that each term in the superpositions constitutes a world in which the objects instantiate the properties corresponding to the eigenvalue of the observable of which they are eigenvectors. With this assumption, we can generate the contradiction:

- By (B) and (*Functional instantiation of properties*) W concludes that, in one world, there is a version of F2 whose system instantiates *spin-up*. In another world, the particle that was measured by F2 instantiates *spin-down*. If W now wonders which version of F2 he shares a world with (what Section 6.1.2.2 called *self-locating uncertainty*), he will reason as follows.
- (*Case 1*) If the version of W (who is uncertain) is in the world in which F2’s system instantiates *spin-up*, then by (A) this is also the same world in which F1’s coin instantiates *tails*. But then, by (C), this is also a world in which A instantiates property *fail*.
- (*Case 2*) If the version of W (who is uncertain) is in the world in which F2’s system instantiates *spin-down*, then by (B) this is also a world in which F1 instantiates outcome *fail*. But then by (D) this is also a world in which A instantiates property *fail*.

Since, in all possible worlds, W concludes that his assistant A instantiates property *fail*, the conjunction of quantum mechanics and (*Functional instantiation of properties*) predicts that whatever world W is uncertain to have ended up in, neither of those could be a world in which the experiment has stopped. But by (E),${\;\mathrm{ok}}_{W}⨂{\mathrm{ok}}_{A}$ has a non-zero probability. Therefore, quantum mechanics implies that there is a non-zero probability that the experiment will stop. Therefore, (*Functional instantiation of properties*) is inconsistent with quantum mechanics.
